# Association between High-Fat Diet during Pregnancy and Heart Weight of the Offspring: A Multivariate and Mediation Analysis

**DOI:** 10.3390/nu14204237

**Published:** 2022-10-11

**Authors:** Wenji Wang, Yu Huo, Jialing Zhang, Da Xu, Fan Bai, Yonghao Gui

**Affiliations:** 1 National Children’s Medical Center, Children’s Hospital, Fudan University, Shanghai 201102, China; 2 National Health Commission (NHC) Key Laboratory of Neonatal Diseases, Fudan University, Shanghai 201102, China; 3 Institute of Pediatrics, Children’s Hospital, Fudan University, Shanghai 201102, China; 4 Cardiovascular Center, Children’s Hospital of Fudan University, Shanghai 201102, China

**Keywords:** DOHaD, high-fat diet, mediation analysis, pregnancy, offspring heart weight

## Abstract

Maternal nutrition and health status in the peri-pregnancy period are closely related to offspring health. Currently, population studies are unable to provide quantitative relationships and effective measures of peri-pregnancy high-fat diet and offspring myocardial remodeling due to the difficulty of obtaining human samples. This study aimed to establish the mouse models of maternal obesity and high-fat diet supplementation and deprivation during pregnancy. The effects of obesity, periconceptional high-fat diet window, fetal weight, sex, and placental weight on myocardial remodeling in the offspring were measured by single-factor and multiple-factor regression analyses. Moreover, the relationship between perinatal high-fat diet/fetal weight and offspring myocardial remodeling was explored using the mediation analysis model. The multivariate analysis showed that the heart weight to body weight (HW/BW) ratio of the offspring decreased by −1.6525 mg/g for every 1-g increase in fetal weight. The offspring HW/BW increased by 1.1967 mg/g if pregnant women were exposed to a high-fat diet throughout pregnancy. The mediation analysis model of a perinatal high-fat diet for the myocardial remodeling of offspring revealed that fetal weight had a suppression effect on the myocardial weight of offspring, accounting for 60.70%; also, it had a mediating effect on the HW/BW of offspring, accounting for 17.10%. Moreover, subgroup analysis showed an interaction between offspring sex and HW/BW in a maternal high-fat diet during pregnancy. Additionally, a quantitative real-time polymerase chain reaction experiment further proved that a perinatal high-fat diet could change the important indicators of myocardial remodeling in offspring. In conclusion, this study found that a high-fat diet in the periconceptional period influenced factors in offspring myocardial remodeling. Moreover, maternal high-fat diet deprivation attenuated the changes in offspring myocardial remodeling. In addition, the role of fetal weight in mediating maternal high-fat diet-mediated offspring myocardial remodeling was quantified. Our study showed that a sensible and healthy diet during the perinatal period, especially during pregnancy, played a positive role in the health of the offspring.

## 1. Introduction

Based on the study of the nutritional status of pregnant women during the famine period, David Barker found that the offspring of pregnant women with nutritional deficiency during pregnancy had a significantly higher incidence of a series of metabolic diseases, such as central vascular disease, abnormal glucose metabolism, hypertension, central obesity, and dyslipidemia, compared with other groups [1,2,3,4]. Hence, he proposed the developmental origins of health and disease (DOHaD) theory, which has updated people’s understanding of disease and health. The first 1000 days of life offer a unique window of opportunity to shape a healthier, more prosperous future for our children, which has drawn continued attention worldwide.

The role of intrauterine programming of the fetus and newborn is closely related to nutrition during pregnancy. At the same time, the dangers of overnutrition on human health are more visible with the gradual improvement of living standards in society. Over the past few decades, the global increase in obesity has been accompanied by a rise in pre-pregnancy obesity [5]. The latest data show that women with obesity during pregnancy already account for 50% of all women of childbearing age [5]. Obesity in pregnant women predicts both short- and long-term adverse health outcomes for the mother and child, and maternal obesity has been found to increase the risk of obesity, diabetes, vascular disease, and heart disease in the offspring [6,7]. The cardiac effects of maternal obesity are characterized by an increased risk of congenital heart disease, with an increase in the prevalence of aortic branch defects, atrial septal defects, and persistent ductus arteriosus with the severity of maternal obesity [8]. In addition, recent evidence shows that the body mass index (BMI) of pregnant women is positively associated with the incidence of cardiovascular disease in early childhood and adulthood [9]. The identification of priority factors before or during pregnancy and the implementation of potentially effective means of prevention and treatment may help curb the worldwide rise in the prevalence of noncommunicable chronic diseases.

Given the current state of the obesity epidemic and its continuing impact on peri-pregnant women and offspring health, particularly cardiovascular health, some studies focused on the molecular mechanisms underlying the mediation of damage to offspring myocardial health caused by maternal high-fat diets and obesity [10,11,12,13,14]. Exposure to maternal obesity-related factors early in life leads to alterations in a range of signaling molecules and metabolic pathways in the offspring myocardium, such as maternal obesity offspring heart transforming growth factor-β (TGF-β), nuclear factor kappa-B (NF-κB), protein kinase B (PKB/AKT), extracellular regulatory protein kinase, and mammalian target of rapamycin (mTOR) activation [10,11,12]. A series of signaling pathway changes trigger energy metabolism disorders and mitochondrial function abnormalities in cardiac myocytes. On the other hand, a maternal high-fat diet can affect the placental health status, leading to placental endothelial cell dysfunction and abnormal adipokine expression [10,13,14], thus causing an increased risk of cardiovascular disease in the offspring. Further, some studies have reported sex-specific differences in offspring cardiac impairment due to a high-fat diet during pregnancy [15]; however, other studies have not reported the same supporting evidence [16,17]. Also, a high-fat diet during pregnancy may cause abnormal birth weight in the offspring, manifesting as overweight or low birth weight infants [18,19], which may be associated with an increased risk of cardiac disease in later life [20]. However, studies have not further confirmed whether low birth weight is a direct or indirect factor in the increased risk of cardiomyopathy in adulthood.

The mechanism for the occurrence and development of maternal high-fat diet and offspring myocardial injury has been described earlier. Cardiac remodeling caused by maternal obesity in offspring has been found to be associated with many factors such as diet [21], placenta [22], sex [3], and fetal weight [23]. However, on the whole, the effect degree of these factors is still unclear. Besides, no specific and quantitative analysis has been performed on the impact of these factors on cardiac remodeling as a whole. Accurate data, such as heart weight and placental weight, are not available in population studies; therefore, it is difficult to conduct this part of the work on the population.

Due to the controllability and consistency of exposure factors and the short life cycle during pregnancy, animal experiments are an important model for studying fetal-borne diseases [24]. The experimental data that are difficult to obtain in population experiments, such as heart weight and placental weight, can be obtained using animal models. These accurate phenotypic data, combined with appropriate statistical methods, can help obtain innovative analysis results and are of guiding significance for pregnancy care.

In this study, a maternal high-fat diet model was established to analyze the influence and mediating effect of a high-fat diet during pregnancy on the heart weight of the offspring. Solid phenotypic data were used to measure the effect degree of high-fat diet time, different window phases on a periconceptional high-fat diet, placenta weight, offspring sex, and offspring body weight on the heart weight, and the ratio of heart weight to body weight (HW/BW). The proportion of the risk factors and the mediation effect of the high-fat animal models on the myocardial weight proportion of offspring were quantitated for the first time. The changes in myocardial reprogramming-related indicators were found to be induced by a high-fat diet during pregnancy. This study provides a more comprehensive perspective on the effects of high-fat exposure in early life on offspring myocardial remodeling and supplies quantitative evidence of the effects of a high-fat diet during pregnancy on the myocardial remodeling of the offspring. In addition, it advocates a reasonable and healthy diet in early life and is of guiding significance for the effect of dietary intervention in perinatal women on offspring health.

## 2. Materials and Methods

### 2.1. Animal Model

The experiments were performed using C57BL/6J mice between 4 and 5 weeks of age, which were purchased from the Experimental Animal Tech Co. of Weitonglihua (Jiaxing, Zhejiang, China). All procedures and animal handling complied with the guidelines of the Shanghai Medical Experimental Animal Care and the 3R principle under the Animal Experiment Committee of the Children’s Hospital of Fudan University (no. 151-2021).

**Figure 1:**
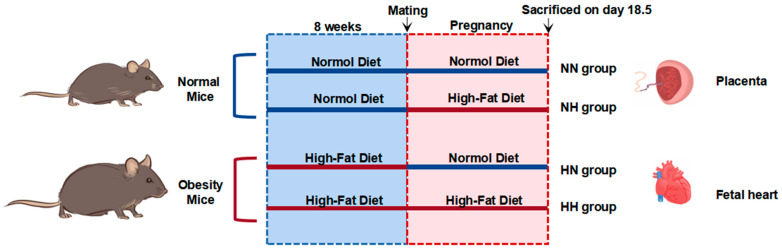
Schematic of the animal model.

The animals were housed under a 12-h light–dark cycle. Twenty mice were randomly divided into four groups of five each after a week of adaptive feeding. Briefly, we gave or did not give a Western diet to mice before and during pregnancy (a well-established obesity model with a high-fat diet, as we described previously [25]). An overview of the experimental design is given in Figure 1. Pregnant mice were subjected to a cesarean section on day 18.5 of gestation. The fetuses and placentas were weighed without membranes and umbilical cords after being carefully dissected from the uterine wall. The tissue samples were flash-frozen in liquid nitrogen and stored at −80 °C after resection.

### 2.2. Extraction of the Placenta and Heart Tissue

On day 18.5 of gestation, parts of the dams were euthanized by isoflurane inhalation. The fetuses, fetal hearts, and placentas were quickly dissected and dried on the medical gauze to remove any remaining fetal membranes, weighed successively, snap-frozen, and stored at −80 °C for further analysis. A piece of fetal tail tissue was collected for sex determination.

### 2.3. Fetal Sex Determination

*Sry* (Sex-determining region Y) gene was used for fetal sex determination as previously described [26,27]. The fetal genotype was determined following the manufacturer’s protocol with the Quick Genotyping Assay Kit for Mouse Tail (Beyotime, D7283M, Shanghai, China). Briefly, 0.5 cm fetal tail tissue and 100 μL of the digestion solution were mixed for genomic DNA extraction. In a similar manner, the tail clippings from an adult male mouse and an adult female mouse were collected, and genomic DNA was extracted for use. The *Sry* gene was amplified by standard polymerase chain reaction (PCR) using forward primer: 5′-TTG TCT AGA GAG CAT GGA GGG CCA T-3′ and reverse primer: 5ʹ-CCA CTC CTCT GT GAC ACT TTA GCC CT-3ʹ. GAPDH was used as an internal reference. The PCR reaction started at 94 °C for 3 min before 35 cycles of 94 °C for 30 s/55 for 30 s/72 for 30 s, followed by a single reaction at 72 94 °C for 30GA GGG CCA T-3′ and reverse primer: 5′-C using 1% agarose-TAE GelRed gel for 40 min at a constant voltage of 80 V. The bands were visualized using a UV light source.

### 2.4. RNA Extraction and Quantitative Real-Time PCR

Total RNA from the heart was extracted using TRIzol reagent (Ambion, Life Technologies, Carlsbad, CA, USA). RNA was reverse transcribed into cDNA using an Evo M-MLV RT Mix Kit with gDNA Clean for qPCR (Accurate Biotechnology, AG11728, Changsha, China) following the manufacturer’s protocol. Quantitative real-time PCR (qRT-PCR) was performed using a Light Cycler 480 real-time PCR system (Roche Applied Science, Basel, Switzerland) and SYBR Green Pro Taq HS Premix PCR kit (AG11701; Accurate Biotechnology, Changsha, China). The sequences of all primers are described in Table S1. PCR reactions were performed in triplicate. All genes were normalized to the housekeeping gene GAPDH, and the changes in the transcript levels of targeted genes were determined using the 2^−∆∆CT^ method.

### 2.5. Statistical Analysis

The data were described and analyzed using SPSS Statistics 26.0 (IBM SPSS Statistics, IBM, Chicago, IL, USA). First, the categorical variables were described using percentages and analyzed using the chi-square analysis to compare differences between rates. For continuous variables, the Shapiro–Wilk test was used to test for normality, and Levene’s test was used to test for the homogeneity of variance. The variables were described as mean ± standard deviation. Student’s *t*-test and one-way analysis of variance were used for comparisons between groups. For variables with a skewed distribution, the median ± interquartile range was used, and further comparisons of the two groups were made using the Wilcoxon signed-rank test. Bonferroni post hoc tests were performed for multiple comparisons. Next, multivariate linear regression was used to analyze the effects of multiple variables on the heart weight/body weight ratio. Heart weight/body weight ratio was used as the dependent variable, and obesity (yes = 1, no = 0), duration of high-fat diet (none = 0, short = 1, medium = 2, long = 3), high fat during pregnancy (yes = 1, no = 0), placental weight (continuous variable), and fetal weight (continuous variable) were used as independent variables. Then, candidate variables were screened by the stepwise regression method. The mediation models were tested using PROCESS v3.5, a macro publicly issued by Andrew Hayes, using 5000 bootstrap samples to test the significance of indirect effects with SPSS software. A *p*-value < 0.05 indicated a statistically significant difference.

## 3. Results

### 3.1. Procedure for the Construction of Animal Models and General Condition of Offspring

First, we randomly divided 20 mice into two groups with no statistically significant difference in initial body weight within each group. Then, the aforementioned two groups of mice were subjected to a normal diet and a high-fat diet for 8 weeks, called the control and obese groups, respectively. Subsequently, the male mice given the normal diet were mated, then given or deprived of the high-fat diet during pregnancy, and finally divided into four groups: NN group, which was always on a normal diet before and during pregnancy; NH group, which was on a normal diet before and a high-fat diet during pregnancy; HN group, which was on a high-fat diet before and a normal diet during pregnancy; and HH group, which was on a high-fat diet before and during pregnancy. The mothers in each group underwent cesarean section on day 18.5 of pregnancy, the placenta and fetal heart tissues were obtained, and the necessary observations were recorded.

We summarized and recorded the general conditions of the offspring fetal mice, and the results are shown in Table 1. The sex of fetal mice was similarly distributed among the groups, and the differences were not statistically significant (*p* = 0.7950 > 0.05). The weight of fetal mice was different in each group. After two comparisons, the weight of fetal mice in the NH, HN, and HH groups was lower than that in the NN group, and the differences were statistically significant (*p* < 0.05). The distribution of fetal heart weight within each group showed that the HN group had the lowest fetal heart weight, which was lower than that of the NN and HH groups (*p* > 0.05). We also obtained the HW/BW ratios. The results showed that the offspring HW/BW values were higher in the NH and HH groups, with the highest HW/BW in the HH group; these values were higher than those in the NN and HN groups, and the differences were statistically significant between the two groups (both *p* < 0.05). We compared the placental weight (PW), and the results showed that the HN group had the highest PW, which was higher than that of the NH and HH groups (both *p* < 0.05). Further, the results showed that the HN and HH groups had the highest placental weight after correcting for the respective fetal weights (PW/BW); the values were higher than those in the NN and NH groups, with statistically significant differences (both, *p* < 0.05).

### 3.2. Analysis of Factors Influencing the Effect of a High-Fat Diet on the Heart Weight of Fetal Mice

We relabeled the information of each group according to sex, high-fat diet during pregnancy, maternal obesity, duration of a high-fat diet, lightness of placental weight, and high or low PW/BW, and performed one-way analysis of the aforementioned factors, to further investigate the influencing factors affecting heart weight and HW/BW ratio. The results are shown in Table 2. The results showed that the heart weight of fetal mice with a high-fat diet during pregnancy, nonobese mothers, and lower offspring PW/BW ratio was lower (all *p* < 0.05). After considering the effect of fetal weight on the heart, we found that only the maternal high-fat diet during pregnancy and the duration of the maternal high-fat diet had an effect on the fetal HW/BW of the offspring. The fetal HW/BW ratio of the offspring with a maternal high-fat diet during pregnancy and a maternal high-fat diet for a long time was higher, and the difference was statistically significant (*p* < 0.0001) compared with that of the offspring with a maternal non-high-fat diet during pregnancy and control offspring.

Maternal obesity, duration of a maternal high-fat diet, maternal high-fat diet during pregnancy, and PW were used as independent variables, and HW/BW ratio was used as the dependent variable. A stepwise regression variable screening method was used to perform a multifactorial linear regression analysis of the effects of maternal factors on offspring fetal HW/BW. The results are shown in Table 3. The multifactorial analysis showed that for every 1-g increase in fetal mouse body weight, the offspring HW/BW decreased by 1.6525 mg/g, and for maternal high-fat food intake during pregnancy, the offspring HW/BW increased by 1.1967 mg/g. Among the independent variables included in the model, the effects of fetal mouse body weight and maternal high-fat food intake during pregnancy on HW/BW ratio were statistically significant (*p* < 0.05).

### 3.3. Analysis of the Mediating Effect of a High-Fat Diet during Pregnancy on the Heart Weight of the Offspring

The multiple linear regression found that a high-fat diet during pregnancy and fetal weight could affect the offspring’s HW/BW ratio. The mediation model of the effect of a high-fat diet during pregnancy on offspring heart weight and HW/BW was established to further investigate the relationship between the factors and whether a high-fat diet during pregnancy influenced offspring heart weight or HW/BW through fetal mouse weight. The variables were assigned as shown in Table 4.

**Figure 2:**
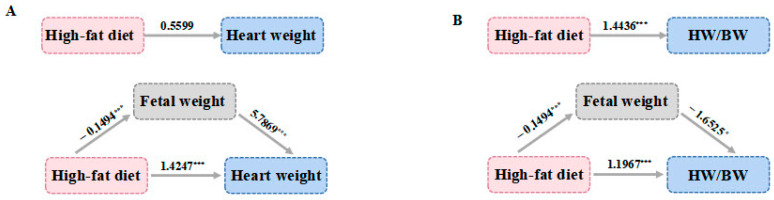
Path models of the mediation analysis between a mother’s high-fat diet during pregnancy and heart weight (**A**) and HW/BW (**B**). “*”, “***”, indicates *p* < 0.05, *p* < 0.001 respectively.

The results of Model 1 in Table 4 showed that the effect coefficient of a high-fat diet during pregnancy and offspring weight was −0.8648, indicating that the mediation effect was the suppression effect, and the proportion of the suppression effect was 60.70%, with 95% CI excluding 0, indicating that the effect was statistically significant. In the direct effect of Model 1, the effect coefficient of a high-fat diet during pregnancy and offspring heart weight was 1.4247, with a statistically significant effect (95% CI: 0.8560–1.9933). The offspring weight showed a positive association with offspring heart weight with a regression coefficient of 5.7869 (*p* < 0.001), as shown in Figure 2A. The coefficient of the effect of a high-fat diet during pregnancy on offspring heart weight was 0.5599 with 95% CI including 0, indicating that the total effect was not statistically significant. As shown in the Model 2 results in Table 4, the effect coefficient of a high-fat diet during pregnancy on the offspring HW/BW ratio was 0.2469, and the proportion of the mediation effect was 17.10% with a statistically significant effect (95% CI: 0.0155–0.5125).

In the direct effect of Model 2, the effect coefficient of a high-fat diet during pregnancy and offspring HW/BW was 1.1967 with a statistically significant effect (95% CI: 0.6936–1.6997). The offspring weight was negatively associated with offspring HW/BW with a regression coefficient of −1.6525 (*p* < 0.05), as shown in Figure 2B. The effect coefficient of a high-fat diet during pregnancy and offspring HW/BW was 1.4436, with 95% CI excluding 0, indicating that the total effect was statistically significant.

### 3.4. Subgroup Analysis of the Effect of Sex on the Effect of a High-Fat Diet during Pregnancy on HW/BW in the Offspring

**Figure 3:**
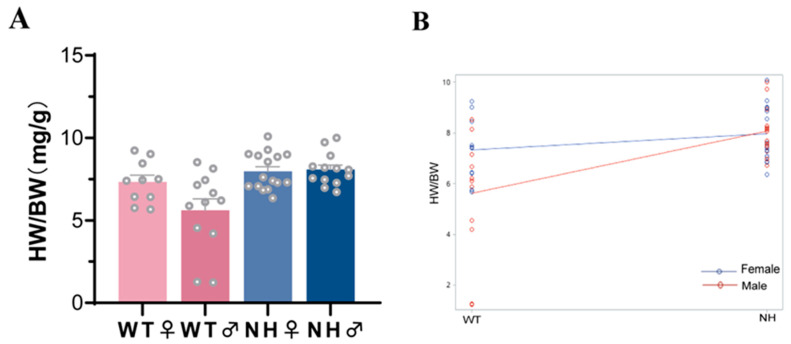
Subgroup analysis of the effect of sex on the effect of a high-fat diet during pregnancy on HW/BW in the offspring. (**A**) Bar graph of HW/BW distribution of different sexes of fetal mice in the WT and NH groups. (**B**) Interaction between offspring sex and the effect of a high-fat diet during pregnancy on HW/BW.

The results of the mediation model analysis indicated a greater direct effect of a high-fat diet during pregnancy on HW/BW. The involvement of the sex factor in the effect of a high-fat diet during pregnancy on HW/BW was further explored by subgroup analysis. In the subgroup analysis, the interaction between sex and the effect of a high-fat diet during pregnancy on HW/BW was observed. As shown in Table 5, the effect of sex alone on HW/BW was not observed (Table 5, *p* = 0.0683 > 0.05), whereas the interaction results suggested an interaction between a high-fat diet during pregnancy and sex (Figure 3 and Table 2, *p* < 0.05). The aforementioned results suggested that a high-fat diet during pregnancy led to male offspring with higher HW/BW.

### 3.5. Effect of a High-Fat Diet on the Expression of Key Indicators of the Fetal Heart in the Offspring

**Figure 4:**
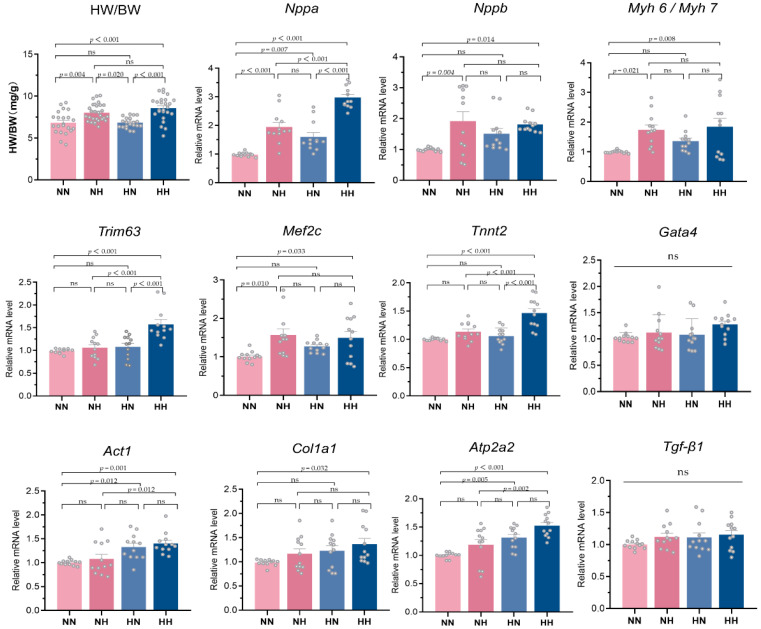
Relative mRNA expression of important indicators of the fetal heart in each group.

We found a significant effect of high-fat dietary intake during pregnancy on offspring HW/BW using the multifactorial regression model and mediated analysis model. The visualized bar graph results and statistical analysis results are shown in Figure 4. For further validation, the qRT-PCR results are also shown in Figure 4. The mRNA expression of myocardial hypertrophy genes *Nppa*, *Nppb*, *Myh 6*/*Myh 7*, and *Mef2c* was significantly higher in the NN and HH groups (*p* < 0.05); *Trim63* and *Tnnt2* mRNA expression was also significantly higher in the HH group (*p* < 0.001), while *Gata4* mRNA expression was not significantly different among the groups (*p* > 0.05). For myocardial fibrosis genes, the mRNA expression of *Col1a1* was significantly higher in the HH group, the mRNA expression of *Act1* and *Atp2a2* was also significantly higher in the HN and HH groups (*p* < 0.05), while the mRNA expression of *Tgf-β1* was not significantly different among the groups (*p* > 0.05).

## 4. Discussion

Strong epidemiological and laboratory evidence shows that maternal overweight and obesity have long-term effects on the health of the offspring [27,28]. Recent evidence suggests that obesity or high-fat dietary intake during pregnancy is associated with the development of heart disease in the offspring [9,29,30]. The molecular mechanisms linking adverse myocardial changes in maternal overweight or obese offspring remain to be fully defined, with the main mechanisms involving genetic factors, intrauterine environment, and placental health [2,9,22,31]. In this study, we constructed animal models of high-fat diet exposure in different stages of perinatal pregnancy, combined with multifactorial analysis, to find the key factors of a maternal high-fat diet leading to offspring myocardial remodeling; examined the effect of offspring weight itself on their myocardial remodeling with the help of mediated analysis models; obtained the direct effect of a high-fat diet during pregnancy on offspring myocardial remodeling, and found the expression of key fetal cardiac genes altered by a high-fat diet during pregnancy.

This study found that the offspring of mothers with a high-fat diet had lower body weight than those in the normal group, regardless of whether the high-fat diet was consumed before or during pregnancy. In recent years, meta-analyses have been conducted on the influence of maternal fat exposure on offspring weight. The results of the forest map showed that a maternal high-fat diet did not affect the birth weight of offspring but increased the weaning weight and final weight of female and male offspring [16]. The finding was not completely consistent with the results of this study, possibly because the high-fat feed components used in different experiments were not the same, leading to inconsistent tolerance of various genera. In addition, the weight of the fetus on day 18.5 of gestation was determined in this study, and the effect of high-fat breast milk alone on fetal weight reprogramming after birth was excluded. The placental weight/fetal weight (PW/BW) of obese mothers was higher than that of nonobese mothers, and the HN group had higher PW/BW levels even when the high-fat diet was resumed during pregnancy. Studies have shown that the dietary supplementation of long-chain polyunsaturated fatty acids (LCPUFA) normalized the maternal LCPUFA level and increased placental LCPUFA accumulation, but did not increase the fetal LCPUFA level. It seemed to indicate that fatty acids were directly proportional to placental accumulation, but were not positively correlated with the fetal content, which might be one of the reasons for the lower fetal weight and higher PW/BW in the HN and HH groups [32].

Thus, HW/BW is usually used as an important indicator to measure cardiac remodeling and hypertrophy [32,33,34,35]. The factors affecting heart weight and HW/BW were analyzed using the single-factor analysis method. The offspring of obese mothers and fetuses with different weight layers of PW/BW had statistically significant differences in fetal cardiac weight, but these differences disappeared after the readjustment for fetal weight. This indicated that fetal weight might be a confounding factor for the influence of obese mothers and PW/BW on offspring HW/BW. Then, combined with multifactorial analysis and based on gradual screening, it was finally found that fetal weight and a high-fat diet during pregnancy were two important factors affecting HW/BW. Some studies have shown that fetal overgrowth and restriction were associated with the changes in the geometry and function of the heart at birth [36]. The association between low birth weight and an increased risk of coronary heart disease has been proved in a large number of different populations [37]. At the same time, some evidence proved the link between maternal obesity or a high-fat diet during pregnancy and heart disease in offspring [9,29,30], which was consistent with the results of this multifactorial analysis. Some studies have shown the effects of fetal weight and maternal obesity or a high-fat diet during pregnancy on the cardiac health of offspring. However, few studies have distinguished the specific effects of fetal weight and maternal obesity or a high-fat diet on cardiac remodeling, especially when abnormal fetal weight and maternal intrauterine nutritional environment coexisted. To solve this problem, we used the mediating effect analysis and made full use of the advantages of animal models to understand the contribution degree of fetal weight and pregnancy high-fat diet to fetal HW/BW, and whether a high-fat diet during pregnancy affected HW/BW by affecting fetal weight. The results showed no difference in the effect of a high-fat diet during pregnancy on heart weight when the dependent variable was fetal heart weight because of the significant masking effect of fetal weight, which masked the direct effect of a high-fat diet during pregnancy on heart weight. Furthermore, for Model 2 of the influence of a high-fat diet during pregnancy on HW/BW, it was found that fetal weight as a mediator accounted only for 17.10% of the mediating effect. Thus, it could be said that the direct effect of a high-fat diet during pregnancy on the change in offspring HW/BW was greater than the mediating effect. In addition, a high-fat diet during pregnancy had a great effect on the myocardial remodeling of offspring, suggesting that the intrauterine environment during pregnancy had a significant impact on offspring health.

The influence of maternal intrauterine exposure to adverse factors on the offspring health of different sexes has always been an important topic in fetal-derived diseases [6,24,25,38,39]. The single-factor analysis, multiple-factor analysis, and mediation effect analysis showed that a high-fat diet during pregnancy had a larger influence on the offspring’s HW/BW. Therefore, the sex subgroup analysis was conducted on the high-fat diet during pregnancy of female offspring. The results showed an interaction between sex and the effect of a high-fat diet during pregnancy on HW/BW and that the male offspring of the mothers with a high-fat diet during pregnancy had higher HW/BW. This finding was consistent with our previous findings [40]. However, this study further demonstrated that the reprogramming effect of a high-fat diet during pregnancy on the offspring’s myocardium occurred earlier in the fetal period by excluding the direct effect of a high-fat diet after birth on the myocardium of mice. Moreover, the age-standardized morbidity and mortality rates for cardiovascular diseases are higher in men than in women [41,42]. The reason why men are more susceptible to the reprogramming of cardiac muscle health early in life is worth exploring in the future.

We used the phenotypic data from the animal models to analyze the models with the help of statistical analysis and found that a high-fat diet during pregnancy had the greatest effect on HW/BW in the offspring. Next, we used qRT-PCR to examine the myocardial-related indicators in fetal mice and found that *Nppa*, *Nppb*, and *Mef2c* were highly expressed in the NH and HH groups. Several studies have shown that the *Nppa* and *Nppb* genes played a role in the development of cardiac hypertrophy and were used as significant markers of cardiac hypertrophy [43,44]. MEF2C is involved in cardiac morphogenesis and myogenesis and regulates the expression of cardiac proteins [45,46,47]. The aforementioned results suggested that a high-fat diet during pregnancy caused alterations in fetal cardiac hypertrophy indicators.

Myocardial contraction in vertebrates is mediated by two molecular motors, *Myh6* and *Myh7*, which are also known as myosin heavy chain α and β, respectively [48]. Relatively subtle changes in the *Myh6/Myh7* ratio can affect cardiac function. Actin-induced ATPase activity of the *Myh7* protein is two to three times lower than that of the *Myh6* protein [49]. The *Myh6/MYh7* ratio was elevated in the NH group versus the HH group in this study, which might be a response to maladaptive cardiac remodeling.

In the present study, the transcript levels of *Cola1* were elevated in the offspring myocardium of mice in the HH group. *Cola1* is the main collagen type in myocardial tissue. *Cola1* expression was elevated in the myocardium of mice on a high-fat diet [50]. Another study indicated that *Cola1* expression was abnormal in the tendon tissue of offspring on a high-fat diet [38]. In combination with the results of the present study, it was likely that a high-fat diet was involved in the regulation and expression of *Cola1*, and its specific mechanism deserves further exploration.

*Atp2a2* plays an important role in cardiomyocyte contraction and diastole and cardiac function, and is a key regulator of Ca^2+^ cycling in cardiomyocytes; its abnormal expression is closely associated with the changes in myocardial contractility [38,51]. Some previous studies have indicated that myocardial injury was synchronized with alterations in a number of indicators, such as cardiac tissue from *Myh6* variant carriers showing significant upregulation of myosin genes, including *Actα1* (actin α1), *Myl2* (myosin light chain 2), *Tnnt2* (cardiac troponin T), and the *Myh6* homolog *Myh7* heavy chain heterodimer encoding β-myosin [52]. Our study also observed changes in other important cardiac-related indicators in the HH group, as well as elevated *Trim63* and *Tnnt2* expression, and upregulation of *Acta1* and *Atp2a2* in the HN and HH groups. We found that myocardial remodeling-related indicators *Nppa*, *Nppb*, *Myh6/Myh7*, *Trim63*, *Mef2c*, and *Tnnt2* were almost the same in the high-fat diet deprivation group during pregnancy as in the control group, even if the mothers were pre-pregnancy obese individuals. In conclusion, we found that the myocardial transcriptional level of the offspring of mothers with a high-fat diet during pregnancy did show alterations in important indicators related to myocardial remodeling. Their myocardial remodeling and hypertrophy during fetal life may indirectly affect their adult myocardial health due to the nonrenewable nature of cardiomyocytes.

## 5. Conclusions

The mouse models of maternal obesity, high-fat diet during pregnancy, and deprivation of high-fat diet during pregnancy were established in this study using animal models. Based on the comprehensive collection and analysis of the placenta, cardiac, and fetal data, the important influencing factors of the effect of the maternal nutrition surplus model on fetal heart weight and HW/BW were identified. Moreover, combined with the strategy analysis and using the mediation analysis model, the effects of a high-fat diet during pregnancy and fetal weight on cardiac remodeling were quantified. In addition, the related indexes of cardiac remodeling induced by a high-fat diet during pregnancy were found. This study demonstrated from a relatively innovative perspective that a high-fat diet during pregnancy, rather than a high-fat diet before pregnancy, was more closely associated with cardiac remodeling in offspring. In this golden window period, dietary intervention in obese individuals could alleviate the elevation of markers such as cardiac hypertrophy in offspring. This study emphasized the importance and long-term impact of diet during pregnancy on the cardiac development of offspring. Furthermore, it also provided the theoretical basis and insight for nutrition intervention during pregnancy for obese and healthy people.

## Figures and Tables

**Table 1:** Characteristics of all pups at day 18.5 of gestation.

	Group					
	NN (*n* = 22)	NH (*n* = 29)	HN (*n* = 21)	HH (*n* = 25)	χ^2^/F	*p* Value
Gender Female Male						
	10 (45.45%)	16 (55.17%)	9 (42.86%)	11 (44.00%)	1.03	0.7950
	12 (54.55%)	13 (44.83%)	12 (57.14%)	14 (56.00%)		
Body Weight (g)	1.31 ± 0.09	1.12 ± 0.13 *	1.08 ± 0.10 *	0.96 ± 0.39 *	40.77	<0.0001
Heart weight (mg)	9.00 ± 1.00	9.00 ± 2.00	7.00 ± 1.00 *^#^	8.00 ± 1.00	16.89	0.0007
HW/BW (mg/g)	6.83 ± 1.35	8.02 ± 1.04 *^△^	6.85 ± 0.63	8.58 ± 1.43 *^△^	13.42	<0.0001
Placenta weight (mg)	93.50 ± 21.00	89.10 ± 9.79	101.00 ± 12.00 ^#^	89.28 ± 14.49 ^△^	17.68	0.0005
PW/BW (mg/g)	73.66 ± 13.01	80.53 ± 6.75	97.68 ± 15.92 *^#^	97.01 ± 25.57 *^#^	26.68	<0.0001

Pairwise comparisons were *Bonferroni* corrected. “*”, “^#^”, “^△^” indicates statistically significant differences compared with the NN, NH, and HN groups, respectively (*p* < 0.05).

**Table 2:** Results of univariate analysis of factors affecting fetal mouse heart weight and HW/BW.

	Heart Weight (M ± Q)	χ^2^	*p*-Value	HW/BW $\left(\overset{¯}{X}\pm S\right)$	t/F Value	*p*-Value
Gender						
Female	8.00 ± 3.00	0.01	0.9795	7.54 ± 1.46	0.79	0.4296
Male	8.00 ± 2.00			7.76 ± 1.26		
High-Fat During Pregnancy						
Yes	8.00 ± 2.00	5.01	0.0251	8.28 ± 1.25	−6.05	<0.0001
No	8.00 ± 2.00			6.84 ± 1.05		
Obesity						
Yes	8.00 ± 1.00	10.61	0.0011	7.79 ± 1.42	−1.03	0.3054
No	9.00 ± 2.00			7.50 ± 1.31		
High-fat diet time						
None	9.00 ± 1.00	16.88	0.0007	6.83 ± 1.35		
Short	9.00 ± 2.00			8.02 ± 1.04	13.42	<0.0001
Medium	7.00 ± 1.00			6.85 ± 0.63		
Long	8.00 ± 1.00			8.58 ± 1.43		
Placenta weight						
Light	8.00 ± 2.00	0.51	0.4732	7.71 ± 1.41	0.52	0.6063
Heavy	8.00 ± 3.00			7.57 ± 1.34		
PW/BW						
Light	9.00 ± 2.00	15.11	0.0001	7.49 ± 1.29	−1.08	0.2844
Heavy	8.00 ± 1.00			7.79 ± 1.44		

**Table 3:** Results of multivariate analysis of factors affecting fetal mouse HW/BW.

Univariate Linear Regression Analysis				
	β	S.E	*t* Value	*p*-Value
Model 1				
Obesity	0.2863	0.2778	1.03	0.3054
Model 2				
High-fat diet time	0.4094	0.1192	3.44	0.0009
Model 3				
High fat during pregnancy	1.4436	0.2386	6.05	<0.0001
Model 4				
Placenta weight	−0.0177	0.0090	−1.96	0.0525
Model 5				
Fetal weight	−2.9170	0.6841	−4.26	<0.0001
**Stepwise linear regression model**				
**Model Total**	**β**	**S.E**	**F**	***p* value**
Fetal weight	−1.6525	0.6738	6.02	0.0160
High fat during pregnancy	1.1967	0.2534	22.30	<0.0001

**Table 4:** Results of mediation analysis of the effect of a mother’s high-fat diet during pregnancy on offspring heart weight and HW/BW.

	Model 1 X (High-Fat Diet) M (Fetal Weight) Y(Heart Weight)		Model 2 X(High-Fat Diet) M (Fetal Weight) Y(HW/BW)	
	Effect Coefficient	95% CI	Effect Coefficient	95% CI
Indirect effect	−0.8648	(−1.3583, −0.4318)	0.2469	(0.0155, 0.5125)
Direct effect	1.4247	(0.8560, 1.9933)	1.1967	(0.6936, 1.6997)
Total effect	0.5599	(−0.0995, 1.2193)	1.4436	(0.9700, 1.9172)
Proportion of suppression/mediated effect	60.70%		17.10%	

**Table 5:** Results of the interaction effect of sex on the effect of a high-fat diet during pregnancy on HW/BW in the offspring.

Factor	*Mean Square*	F Value	*p* Value
NH	29.88	12.97	0.0008
Gender	8.02	3.48	0.0683
NH*Gender	10.36	4.49	0.0393

## Data Availability

Not applicable.

## Supplementary Materials

The following supporting information can be downloaded at: https://www.mdpi.com/article/10.3390/nu14204237/s1, Table S1. Information of primers for real-time RT-PCR.
