# Supplementary material for: Association between High-Fat Diet during Pregnancy and Heart Weight of the Offspring: A Multivariate and Mediation Analysis
Source: Nutrients. 2022 Oct 11;14(20):4237. doi: 10.3390/nu14204237 (PMC9609645; doi:10.3390/nu14204237)
Supplement: Supplementary file 1 [file nutrients-14-04237-s001.zip › nutrients-1901478-supplementary.pdf]

**Table S1.** Information of primers for real-time RT-PCR.

| mRNA | Sequence (5′-3′) |
|---|---|
| Nppa | F:TACAGTGCGGTGTCCAACACAG |
| | R:TGCTTCCTCAGTCTGCTCACTC |
| *Nppb* | F:TCCTAGCCAGTCTCCAGAGCAA |
| | R:GGTCCTTCAAGAGCTGTCTCTG |
| *Myh6* | F:GCTGGAAGATGAGTGCTCAGAG |
| | R:CCAGCCATCTCCTCTGTTAGGT |
| *Myh7* | F:GCTGGAAGATGAGTGCTCAGAG |
| | R:TCCAAACCAGCCATCTCCTCTG |
| *Trim63* | F:TACCAAGCCTGTGGTCATCCTG |
| | R:ACGGAAACGACCTCCAGACATG |
| *Mef2c* | F:GTGGTTTCCGTAGCAACTCCTAC |
| | R:GGCAGTGTTGAAGCCAGACAGA |
| *Tnnt2* | F:GCTACAGACTCTGATCGAGGCT |
| | R:GCTCATTGCGAATACGCTGCTG |
| *Gata4* | F:GCCTCTATCACAAGATGAACGGC |
| | R:TACAGGCTCACCCTCGGCATTA |
| *Acta1* | F:ACCATCGGCAATGAGCGTTTCC |
| | R:GCTGTTGTAGGTGGTCTCATGG |
| *Col1α1* | F:CCTCAGGGTATTGCTGGACAAC |
| | R:CAGAAGGACCTTGTTTGCCAGG |
| *Atp2α2* | F:GTGAAGTGCCATCAGTATGACGG |
| | R:GTGAGAGCAGTCTCGGTAGCTT |
| *Tgf-β1* | F:TGATACGCCTGAGTGGCTGTCT |
| | R:CACAAGAGCAGTGAGCGCTGAA |
| *Gapdh* | F:CATCACTGCCACCCAGAAGACTG |
| | R:ATGCCAGTGAGCTTCCCGTTCAG |
